# Apoptosis of Rheumatoid Arthritis Fibroblast-Like Synoviocytes: Possible Roles of Nitric Oxide and the Thioredoxin 1

**DOI:** 10.1155/2013/953462

**Published:** 2013-04-03

**Authors:** Huili Li, Ajun Wan

**Affiliations:** ^1^School of Pharmacy, Shanghai Jiao Tong University, Shanghai 200240, China; ^2^School of Chemistry & Chemical Technology, Shanghai Jiao Tong University, Shanghai 200240, China

## Abstract

Rheumatoid arthritis is a chronic inflammatory disease characterized by synovial hyperplasia and progressive joint destruction. The impaired apoptosis of rheumatoid arthritis fibroblast-like synoviocytes (RA-FLS) is pivotal in this process. However, the molecular mechanisms responsible for the reduced apoptosis are not fully understood. Both nitric oxide and thioredoxin 1 as two important mediators are widely investigated in the pathogenesis of rheumatoid arthritis. Interestingly, studies have showed that thioredoxin 1 may serve as a master regulator of S-nitrosylation of caspase-3 to fine-tune apoptosis *in vivo*. Thus, it is anticipated that further investigations on the role of thioredoxin 1 in the S-nitrosylation and denitrosylation of caspase-3 in RA-FLS will likely provide a novel understanding of mechanisms implicated in the impaired apoptosis of RA-FLS. In this paper, we will provide an overview on pathways involved in the reduced apoptosis of RA-FLS and then discuss specially the possible roles of nitric oxide and the thioredoxin 1 redox system associated with apoptosis of RA-FLS.

## 1. Introduction 

Rheumatoid arthritis (RA) is a chronic disease of complex causes that primarily affects joint structure and function, ultimately leading to joint destruction. One of the important pathophysiological aspects of RA is a substantial increase in the number of resident synovial cells, which are of mesenchymal origin due to their phenotypic appearance as well as cellular characteristics and have been called fibroblast-like synoviocytes (FLS). Rheumatoid arthritis fibroblast-like synoviocytes (RA-FLS) play pivotal roles both in the initiation and the perpetuation of RA. These cells have been linked most prominently to the progressive destruction of articular structures, particularly cartilage [1, 2]. 

RA-FLS are resistant to programmed cell death (apoptosis) induced by the apoptotic stimulus signals. Multiple mechanisms may contribute to the reduced apoptosis. Since RA is a chronic inflammatory disease characterized by synovial hyperplasia and progressive joint destruction, both nitric oxide (NO) and the thioredoxin 1 (Trx1) as two important mediators are widely investigated in the pathogenesis of RA. However, their direct roles in the regulation of RA-FLS remain unclear. In this paper, we provide an overview on pathways involved in the reduced apoptosis of RA-FLS and then discuss specially the possible roles of NO and the Trx1 redox system.

## 2. The Apoptotic Pathways in RA-FLS

RA-FLS are one of the dominant cell types in the terminal layer of the hyperplastic rheumatoid synovium. At the molecular level, synovial fibroblasts are characterized by the activation of signaling cascades that result in the inhibition of apoptosis [1–4]. Furthermore, impaired apoptosis may contribute to the accumulation of RA-FLS and RA-FLS-mediated disease progression [1–4]. 

Apoptosis is a mechanism by which cells undergo death to control cell proliferation or in response to DNA damage. Apoptosis occurs through two main pathways: the extrinsic pathway triggered by ligands binding to cell death receptors, the intrinsic or mitochondrial pathway initiated by genotoxic stress. Apoptosis is tightly regulated by a complex regulatory network to maintain homeostasis. Mutual interactions exist in both pathways and they converge to a final common pathway, involving the activation of a cascade of proteases called caspases leading to apoptosis.

Recent evidences suggest that RA-FLS exhibit alterations in mitochondrial pathways of apoptosis and are resistant to receptor-mediated apoptosis at multiple levels. Some of the key pathways are described in the following section.

### 2.1. The Intrinsic Pathway

One of the most important regulators of the intrinsic pathways is the Bcl-2 family of proteins. Recent studies suggest a significant role for Bcl-2 family proteins in inhibiting the cell death of RA-FLS. Perlman et al. have demonstrated increased expression of Bcl-2 in RA-FLS associated directly with the extent of synovial lining thickening in situ [5]. Kurowska and colleagues have shown that the inflammatory cytokine IL-15 increased Bcl-2 and Bcl-xL mRNA levels [6]. In addition, Mcl-1, an antiapoptotic member of the Bcl-2 family, was also induced in RA-FLS, which contributed to the resistance of RA-FLS against apoptosis [7]. 

### 2.2. The Extrinsic Pathway 

Activation of the extrinsic pathway is initiated by the ligation of specific death receptors with the corresponding ligands. RA-FLS are relatively resistant to receptor-mediated apoptosis at multiple levels although RA-FLS have been demonstrated to express a variety of death inducing surface receptors of the tumor-necrosis-factor-(TNF-s) receptor family. For example, Fas gene was constitutively expressed by cultured FLS; however, engaging this molecule on RA-FLS only killed 15–20% of cells [8]. The group of Mountz demonstrated that TNF*α* can induce apoptosis in RA-FLS only, when NF-*κ*B was inhibited [9]. Although the data for TRAIL-triggered apoptosis have been conflicting, most data suggested that the susceptibility of RA-FLS to TRAIL-induced apoptosis was somewhat lower than that to FasL/CD95L and largely depending on the cell cycle [10]. The mechanisms underlying the resistant of RA-FLS to receptor-mediated apoptosis are not completely understood, but prominent findings are that the inhibition of apoptosis is associated also with a number of soluble factors such as soluble Fas/CD95-ligand (sFasL/sCD95L), and the soluble decoy receptors DcR3. In RA patients, the concentration of sFasL was remarkably higher, which can antagonize Fas signalling [11]. Hayashi and colleagues reported that the expression of the DcR3 prevented RA-FLS from Fas/CD95-induced apoptosis [12]. Of interest, TNF*α* increased release of sFasL and induced the expression of DcR3 [12, 13]. This provides other mechanisms by which TNF*α* interferes with Fas/Fas-ligand-mediated apoptosis.

Of the tumor suppressor genes implicated in RA, the p53 tumor suppressor is especially important. It is known that the p53 tumor suppressor induces cell growth arrest or apoptosis. The balance between p53-upregulated modulator of apoptosis (PUMA) and the cell-cycle-regulating gene p21WAF/CIP1 (p21) determines the onset of arrest, or death, in response to exogenous p53 expression [14]. In general, exposure to cellular stress can trigger the p53 tumor suppressor, which leads to cell-cycle arrest through p21 and subsequent repair. However, in the scenario of excessive cellular damage or disruption of the arrest response, PUMA and NOXA genes are upregulated, and this ultimately results in apoptosis. In rheumatoid arthritis synovium, the apoptosis is relatively lacking although p53 expression is elevated in the rheumatoid intimal lining [15]. The two possible reasons are mutations in p53 and the relatively inability of wildtype p53 to induce PUMA in synoviocytes. Indeed, p53 mutations have been found in RA synovium, which were functionally relevant as demonstrated by their dominant negative activity in cell transfection experiments [16, 17]. In addition, expression of PUMA was surprisingly low in RA-FLS, and disruption of PUMA provided another explanation for the lack of p53-induced FLS apoptosis [18].

In addition to external signals, modulation of pathways downstream of death receptors by intrinsic molecules has also been studied in RA-FLS. One of the molecules that have been suggested to be involved is pro-caspase-8/FLICE-like inhibitory protein (FLIP). Increased expression of FLIP induced by TNF*α* via the NF-*κ*B pathway contributed to the resistance of RA-FLS to FasL/CD95L-induced apoptosis [19]. It is noteworthy to mention that posttranslational modification (PTM) of signaling proteins also plays a role in RA-FLS apoptosis. An example is the small ubiquitin-like modifier (SUMO) family of proteins. Pap et al. demonstrated that elevated expression of SUMO-1 in RA-FLS enhanced SUMOylation of nuclear PML protein. This PTM of PML protein inhibited Fas-mediated apoptosis in RA-FLS through nuclear trapping of proapoptotic molecules such as DAXX [20, 21].

## 3. Possible Role of NO in the Reduced Apoptosis of RA-FLS

NO is an endogenously produced small molecule that has critical roles in cellular signaling and is involved in a variety of physiological processes. Research over the past couple of decades suggests that NO can have opposite biological effects, depending upon the level of NO induction, the temporospatial mode of NO action, intracellular targets of NO, and other environmental and pathophysiological conditions. The redox state and chemistry of NO facilitate its interaction with various proteins and thus regulate diverse intracellular and intercellular signaling events. 

NO has also emerged as an important mediator in the collagen-induced arthritis and RA synovium. NO levels are increased in synovial fluid in RA. NO mediates many different cell functions at the site of synovial inflammation, including cytokine production, signal transduction, mitochondrial functions, and apoptosis [22–30]. In addition to these well-established mechanisms, by which NO contributes to the pathophysiology of rheumatoid arthritis, the role of NO in modulating of the apoptosis-inducing signal pathway has also been investigated. Multiple mechanisms have been identified by which NO may regulate apoptosis [31–40]. Recently, S-nitrosylation of pro- and antiapoptotic proteins has begun to receive increasing attention and has important roles in the regulation of apoptosis by NO. Migita et al. reported that the NO donor inhibited Fas-induced caspase-3 activation in rheumatoid synovial cells [41]. In their experiment, NO donor neither affected the Fas expression nor interrupted Fas-induced caspase-8 cleavage or subsequent cytochrome c releasing into the cytosol in rheumatoid synovial cells. However, NO donor suppressed the proteolytic processing and activation of caspase-3 in Fas-treated synovial cells. Based on these observations, it has, therefore, been hypothesized that NO may inhibit the activation of caspase-3 by *S*-nitrosylation of the catalytic cysteine residue, and the *S*-nitrosylated-caspase-3 (SNO-caspase-3) cannot be proteolytically cleaved for subsequent activation of apoptosis. 

Caspase-3 is a central executioner caspase in the apoptotic signaling pathway. Although diverse upstream signaling pathways can regulate activation of caspase-3, a wealth of evidence supports that the reversible *S*-nitrosylation in caspase-3 plays an important role. 

*S*-nitrosylation is the modification of a cysteine thiol on a protein or peptide by an NO group and formation of *S*-nitrosothiols (collectively referred to as SNOs) [42]. Biological *S*-nitrosylation can take place by transnitrosylation, which involves the transfer of an NO group from a cysteine in one protein to a specific cysteine in another protein [43]. In contrast, denitrosylation is defined as the removal of NO groups primarily from the thiol side chain of cysteines in SNOs. Studies have also identified that denitrosylation proceeds nonenzymatically or enzymatically [44].

It is not well understood how the levels and dynamic change of SNO proteins are determined *in vivo* or in diseases. However, it is clear that this change depends on the rates of both S-nitrosylation and denitrosylation of a specific protein. With regard to the reversible S-nitrosylation in caspase-3, Trx redox system has gained much attention because it can either transnitrosylate or denitrosylate caspase-3 [45–48].

## 4. Redox PTMs of the Trx1 System Regulate S-Nitrosylation and Denitrosylation of Caspase-3

The Trx1 system consists of redox active Trx1, thioredoxin reductase (TrxR1), and reduced NADPH. Trx1 is a cytosolic and extracellular enzyme [49, 50]. TrxR1 is a selenoenzyme in the cytosol [49, 50]. NADPH is derived mainly from cellular metabolism.

Mitchell and Marletta first demonstrated that a specific transnitrosation reaction between procaspase-3 and thioredoxin-1 occured in cultured human T cells and that this interaction was critical to caspase-3 activity and apoptosis [45]. Stoyanovsky and coworkers first reported that the reduced or dithiol form Trx (Trx-(SH)_2_) catalyzed the denitrosylation of SNO-caspase-3 in chemical systems [47]. Benhar et al. further identified cellular Trx/TrxR system regulated basal and stimulus-induced denitrosylation of SNO-capsase 3 [48]. Collectively, the extent of *S*-nitrosylation of caspase-3 appears to reflect the balance between the addition and removal of NO groups in caspase-3. Both *S*-nitrosylation and denitrosylation of caspase-3 via Trx1 cooperate, which may be a major means for Trx1 to fine-tune apoptosis. 

**Figure 1:**
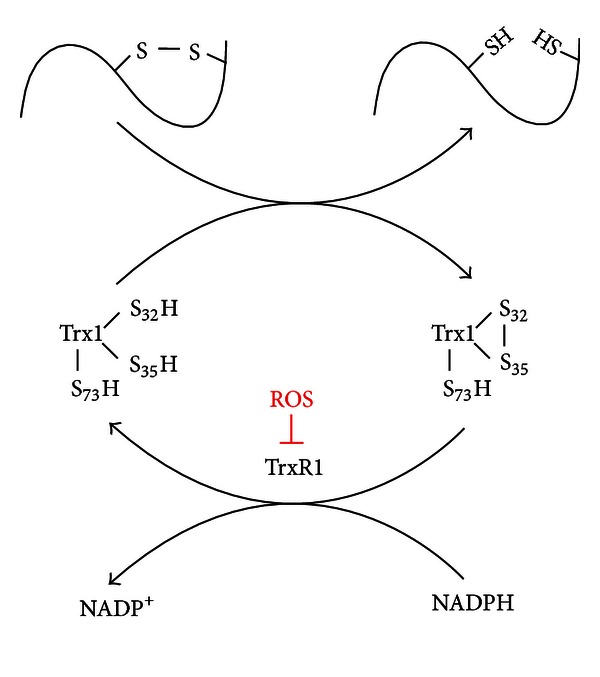
The proposed pathway for the accumulation of Trx-S_2_ in RA-FLS. Under normal conditions, Trx1-(SH)_2_ reduces disulfide bonds in its substrates to form free thiols, while Trx1-(SH)_2_ is oxidized to Trx1-S_2_ simultaneously. TrxR1 further converts the disulfide bonds in Trx1-S_2_ to Trx1-(SH)_2_ in the presence of NADPH. However, under elevated reactive oxygen species (ROS) in RA-FLS, the oxidized form of TrxR1 is accumulated, which reduces the activity of TrxR1 and causes the accumulation of Trx1-S_2_ in RA-FLS.

Trx1 from all organisms contains a conserved active site sequence Cys-Gly-Pro-Cys with two active cysteines (Cys32 and Cys35) that are essential for its functions as protein disulfide oxidoreductase. The Trx-(SH)_2_ can utilize its exposed nucleophilic Cys32 residue to attack the disulfide in the target protein and form a transient mixed disulfide. This mixed disulfide is subsequently attacked by the Cys35 residue to generate the oxidized Trx1 (Trx1-S_2_), and subsequently the disulfide in the target protein is reduced simultaneously. The disulfide presented in the active sites of Trx-S_2_ has to be reduced to a dithiol form to exhibit its disulfide reductase activity. This reduction is carried out by TrxR using NADPH as a reductant (Figure 1) [49, 50]. In addition, human cytosolic Trx1 contains additional structural cysteines, Cys62, Cys69, and Cys73, outside its active site sequence. These cysteines can be modified in a variety of ways under oxidative or nitrosative stress, affecting both its structure and catalytic activity [51–55].

It is generally thought that redox status of the active sites Cys32 and Cys35 appears to be crucial regulators. The free thiol in Cys32 of Trx1-(SH)_2_ is the prerequisite and responsible for Trx1 as a denitrosylase [47, 48, 51–53, 56]. On the contrary, only the Trx1-S_2_, which contains an intramolecular disulfide bond connecting Cys32 and Cys35, could be nitrosylated on Cys73 which is responsible for transnitrosylating activity of SNO-Trx1, including SNO-caspase-3 [51–55].

In addition, the subcellular localization and stability of the SNO-Trx are also tightly regulated by different reductants, such as reduced Trx1 and glutathione (GSH), in cells. For example, the denitrosylation of SNO-Trx can also occur in the presence of either Trx-(SH)_2_ or GSH [52, 56]. However, SNO-caspase-3 is relatively stable in the presence of 5–10 mM GSH [57].

## 5. Could RA Set the Stage for the Formation of SNO-Trx1 and SNO-Caspase-3 in RA-FLS?

Like NO, Trx1 has multiple biological activities in regulation of apoptosis. The mechanisms responsible for these activities have not been completely established. Is redox PTM of Trx1 one of explanations for its bioregulator of apoptosis? In the following section, we will focus on the understanding of redox PTM of Trx1 in RA and suggest a novel insight for future studies. 

It is proposed that reactive oxygen species (ROS) can initiate a wide range of oxidative reactions which are likely to play a role in the pathophysiology of RA through self-perpetuating cycle of inflammation and destruction. It has recently been well reviewed [28, 30, 58]. However, the relationship between ROS and apoptosis of RA-FLS has not yet been discussed specifically. With certainty, it would be impossible to understand the regulatory mechanisms of ROS in the apoptosis of RA-FLS without considering the complex signaling pathways of ROS. However, the discussions below aim to be focused specifically on the function and regulation of redox PTM of Trx1 in RA. 

It has been reported that Trx1 levels were elevated in the synovial tissue, fluid, and serum of RA patients [59–61]. It was also observed that TrxR1 activity was lower in the serum of RA patients [62, 63]. In addition, the activation of intracellular oxidative stress in RA-FLS has also been reported. Particularly with respect to the regulation of the Trx1/TrxR1 system, studies with cultured RA-FLS have demonstrated an alteration of the Trx1/TrxR1 system, that could be one potential explanation for the causes of oxidative stress related to RA disease [59, 63]. For example, Maurice and colleagues first reported that exogenous oxidative stress (H_2_O_2_) or inflammatory cytokine (TNFa) induced an increase in the expression of Trx in RA-FLS [59]. Lemarechal et al. found that H_2_O_2_ also caused a time-dependent accumulation of oxidized TrxR1, which reduced TrxR1 activity in RA synoviocytes [63]. 

It is well established that the Trx1 system is the most important constituent of the intracellular redox milieu controlling the redox state and the redox regulation of cellular processes. The thiol/disulfide redox state of redox-sensitive proteins is maintained at the expense of the formation of the disulfide in the active sites of Trx-S_2_ [64]. TrxR1 is the only enzyme which catalyzes the NADPH-dependent reduction of a redox-active disulfide in Trx1 [64, 65]. Regrettably, a characteristic of TrxR1 is its sensitivity to oxidizing conditions, leading to a change in conformation and reduction in activity. Lemarechal et al. [63] found that TrxR1 activity decreased although TrxR1 mRNA and protein expression levels markedly increased in RA synoviocytes after H_2_O_2_ and superoxide treatment. They further identified that exogenous oxidative stress induced the formation of carbonyl groups in TrxR1 protein and oxidized the selenocysteine of the active site and caused a time-dependent accumulation of oxidized TrxR1. Furthermore, they demonstrated that the oxidation of TrxR1 was irreversible by the addition of NADPH in RA cells incubated for long periods. In addition to direct oxidation inactivation, TrxR1 activity seems to be modulated by a cell-specific intricate pattern [66]. It is well known that mammalian TrxR1 has a selenocysteine residue within a conserved C-terminal GCUG motif that is essential for its Trx-reducing activity. Numerous experimental results suggested that the expression or activity of TrxR1 is dependent on an adequate selenium supply. Higashikubo first reported that TrxR activity decreased in kidney and liver in rats fed a selenium-deficient diet [67]. Lu and coworkers identified that a low-activity form of TrxR in which selenocysteine residue was replaced by cysteine in liver from selenium-deficient rat [68]. With regard to RA, the plasma and synovial fluid concentrations of selenium may be altered due to the increased proinflammatory and immunoregulatory cytokines in the setting of active RA. Several investigators have reported depressed plasma or synovial fluid selenium values in RA patients compared with those in the healthy and osteoartritis patients [69]. Considering the metabolically active nature of RA-FLS, a decrease of cellular TrxR activity can be expected. Thus, it might be speculated that Trx1 was maintained in an oxidized form and could not be restored to a reduced form for its denitrosylase or reductase activity, due to the consequence of TrxR1 inactivation or a low activity under RA-FLS environment (Figure 1). 

It is important to note that the upregulation of NOSs can also take place, resulting in an increase of NO in RA-FLS [26]. With the many known data that Trx-S_2_ can be nitrosylated at Cys73 to form SNO-Trx1 following nitrosative stress [51–55], it is also possible that cytosolic SNO-Trx1 accumulates in RA-FLS. It was reported that SNO-Trx1 is not the substrate of TrxR1, which suggests that SNO-Trx1 might become “uncoupled” from TrxR1, allowing it to engage in NO group transfer reactions rather than in denitrosylation or reduction processes [55]. Although both reduced GSH and Trx1 are able to make SNO-Trx1 denitrosylated, their ability of denitrosylation might be insufficient due to the depletion of GSH in cells induced by severe and/or chronic oxidative stress from RA [52, 70]. For example, levels of surface thiols and GSH of leukocytes from RA patients are significantly lower than those of leukocytes from controls [71]. 

**Figure 2:**
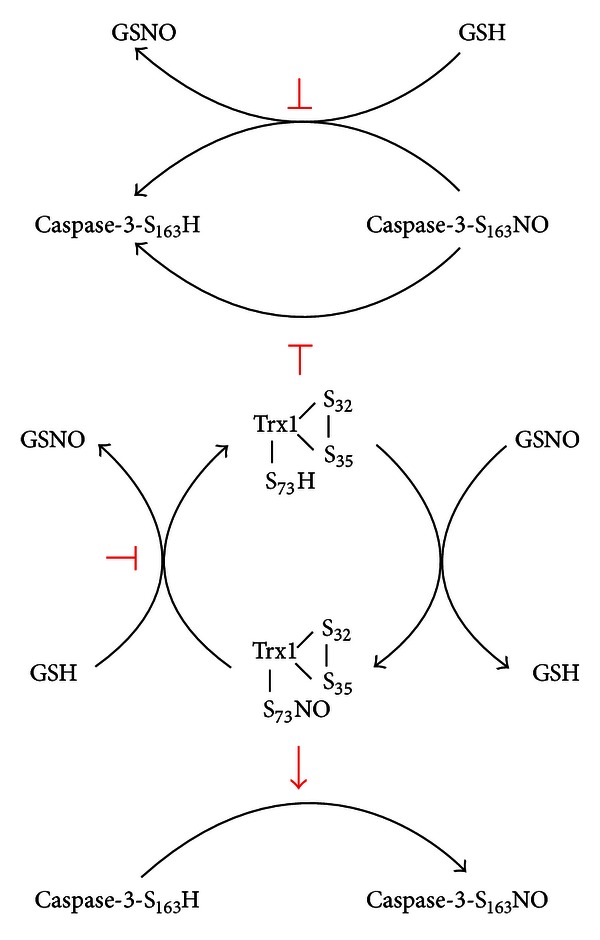
The proposed mechanism for the formation of hyper-S-nitrosylation of caspase-3. Under nitrosative stress, Trx1-S_2_ can be nitrosylated to SNO-Trx1-S_2_, which further transnitrosylates caspase-3 to SNO-caspase-3. Both SNO-caspase-3 and SNO-Trx1-S_2_ may become insufficiently denitrosylated by GSH and Trx1-(SH)_2_ due to their depletion under the oxidative stress in RA-FLS, which further enhances the accumulation of SNO-caspase-3 and SNO-Trx1-S_2_.

SNO-Trx1 may serve the following two purposes: increase its ability to transnitrosylate caspase-3 and lose its ability to denitrosylate SNO-caspase-3, which results in a steady-state increase in S-nitrosylation of caspase-3 (Figure 2). Collectively, one might envision whether there is a steady-state hyper-S-nitrosylation of caspase-3 in RA-FLS, which could contribute to its impaired apoptosis. Regrettably, direct evidence in support of the hypothesis above has not been obtained.

It is worth noting that the relationship between the dysfunctions of Trx/TrxR system and RA-FLS survival is complex due to the following reasons. Firstly, Trx1 has a wide variety of biological activities, such as antioxidant, growth control, and antiapoptotic properties resulting from interaction with target molecules [49, 50]. With regard to the role of Trx1-mediated S-nitrosylation in regulating apoptosis, S-nitrosylation of apoptotic regulatory proteins has both positive and negative regulatory roles to allow cells to fine-tune their response to apoptotic signals [40]. Furthermore, Kabuyama and coworkers [72] examined the gene expression profiles of synovial cells from patients with RA and found the upregulation of TrxR1 but not Trx1 mRNA. They further demonstrated that TrxR1 inhibits apoptosis induced by self-generated ROS. These findings suggest that TrxR1 function as an antiapoptotic factor independent of Trx1.

## 6. Conclusions and Perspectives

It is clear that RA-FLS exhibit alterations in mitochondrial pathways of apoptosis and are resistant to receptor-mediated apoptosis at multiple levels. Multiple mechanisms may contribute to this reduced apoptosis. Recently, accumulating evidence has suggested that the cellular redox state is dysregulated and plays an important role in driving and possibly initiating RA. An important regulator of the redox state of proteins is Trx/TrxR system.

The functional roles of Trx1 in regulating cellular apoptosis are multifaceted. On the one hand, reduced Trx1 can exert its antiapoptotic effect by directly functioning as an antioxidant, ASK-1 inhibitor, a denitrosylase for SNO-caspase-3. On the other hand, SNO-Trx1-mediated transnitrosation of caspase-3 may be partially responsible for the inhibition of apoptosis [51–55]. It seems possible that cross-regulatory mechanism of signaling pathways leading to synovial hyperplasia depends on various stages of RA progression and the context of the signals RA-FLS receives. Thus, it is anticipated that further investigations on the role of Trx/TrxR system in relation to biologically relevant SNOs will likely provide a novel understanding of mechanisms implicated in the impaired apoptosis of RA-FLS. To analysis and address this conceptual model, there is an urgent need to investigate how NO, Trx1, TrxR1, caspase-3, and others are regulated inside or outside of synoviocytes (cytosol or mitochondria) during the development of RA. Much work is needed to more precisely define the different modifications of apoptotic regulatory proteins and their functional implications in RA-FLS. For example, which part of the SNO-proteome changes is the most important contributor to the pathology of RA and possible therapeutic interventions? How about the subcellular localization of SNO-proteome in RA? Are there defects in the S-nitrosylation and denitrosylases pathway in RA-FLS and RA synovium in situ? 

However, we are facing mainly an analytical problem. Although we can identify PTMs of a lot of proteins using proteomic analyses, the current proteomic approaches are not sensitive enough to detect low abundant apoptotic regulatory molecules. In addition, a subcellular localization study is another challenge. Developing new sensitive mass spectrometry detection approaches coupled with biochemical approaches could provide deeper insight into the mechanisms and roles of Trx1/TrxR1 system in the impaired apoptosis of RA-FLS.
